# Increased incidence of vertebral fractures in German adults from 2009 to 2019 and the analysis of secondary diagnoses, treatment, costs, and in-hospital mortality

**DOI:** 10.1038/s41598-023-31654-0

**Published:** 2023-04-28

**Authors:** Siegmund Lang, Nike Walter, Viola Freigang, Carsten Neumann, Markus Loibl, Volker Alt, Markus Rupp

**Affiliations:** 1 Department for Trauma Surgery, University Medical Center Regensburg, Franz-Josef-Strauss-Allee 11, 93053 Regensburg, Germany; 2 Department of Spine Surgery, Schulthess Clinic Zurich, Lenghalde 2, 8008 Zurich, Switzerland

**Keywords:** Epidemiology, Trauma, Fracture repair, Health care economics, Epidemiology

## Abstract

The aim of this cross-sectional study was to present the nationwide rates of hospitalized patients with vertebral fractures over one decade and to comprehensively analyze the treatment characteristics and direct costs incurred in 2019. Therefore, the trends in the incidence rate were quantified based on annual ICD-10 diagnosis codes from all German medical facilities between 2009 and 2019, provided by the Federal Statistical Office (Destatis). The ICD-10 Codes “S12.0-2; S22.0-; S32.0-, and S32.1-2” were evaluated. The relative change from 2009 through 2019 was determined. Using data from the Institute for Hospital Remuneration Systems (InEK) for 2019 the secondary diagnoses, OPS-codes, intensive care unit (ICU) treatment, in-hospital mortality, the proportion of G-DRGs and cumulative costs were evaluated. The documented number of vertebral fractures increased by 45.6% between 2009 and 2019 to an incidence of 150.7 per 100,000 inhabitants. The lumbar spine was most commonly affected with an incidence of 70.5/100,000 inhabitants in 2019 (46.8% of all vertebral fractures). The highest increases were seen in the numbers of subaxial cervical fractures (+ 121.2%) and sacral fractures (+ 306.6%). Of all vertebral fractures in 2019, 63.7% were diagnosed in women and 69.0% in patients aged 70 years or older. Osteoporosis was documented in 17.9% of cases as a concomitant diagnosis. In 10.1% of all cases, an ICU treatment was documented. The in-hospital mortality was 2.0% in 2019. I68D was the most frequently used G-DRG code, accounting for 33.3% of cases. The total direct costs for inpatient treatment in 2019 amounted to €589,205,715. The evaluation of 955,091 vertebral fractures showed a sharp increase in the nation-wide incidence rate. The presented age and sex distribution, the comorbidity profile and the in-hospital mortality rate indicate the importance of comprehensive geriatric assessment and emphasize the need for spinal care centers to be established.

## Introduction

Injuries to the spine account for a relevant proportion of trauma patients, with thoracolumbar fractures being among the most severe injuries of the human skeleton^1,2^. Cervical spine injuries are observed in approximately 2–3% of all accident victims^3,4^. The most common cause of traumatic spinal injury are traffic accidents, followed by falls from low and high heights (> 2 m)^5,6^. The Global burden of Disease study 2019 revealed an worldwide increase of fractures of the vertebral column of 37.7% between 1990 and 2019^7^. Spinal fractures have been reported to be associated with spinal cord injuries (SCI) in up to 20% of cases, especially for injuries to the lower cervical spine and upper to mid-thoracic spine^6^. The quality of life of patients with thoracolumbar fractures is reported to be impaired, typically independent of the treatment modalities but correlated with the severity of the injury^8–10^. While many patients who sustain a vertebral fracture can be treated as outpatients, a considerable number require impatient care, which is associated with significant costs. Despite this, current knowledge of the epidemiology of vertebral fractures is largely based on relatively small populations, and there are few nation-wide analyses^2,11^. The effect of geriatric injuries and osteoporotic fractures on the prevalence of vertebral fractures is not well understood. It is recognized that vertebral fractures are among the most common skeletal fractures associated with low bone mass and other causes of skeletal fragility^12^. Country-specific epidemiological analyses are essential as the incidence, hospital care structure and reimbursement systems vary from country to country: Discharge rates of hospitalized patients with vertebral fractures can vary by more than fourfold between European countries^13^. Detailed analyses of epidemiologic data, current treatment, and incurred direct costs are an essential resource for healthcare systems stakeholders and provide valuable insights into the impact of current prevention and treatment strategies.

Therefore, this study aims (1) to determine trends in the nationwide incidence rate of vertebral fractures for Germany from 2009 to 2019 by age, sex, and anatomic localization. Second (2), to provide a comprehensive overview of the treatment characteristics based on documented OPS codes, comorbidities, concomitant diagnoses, in-hospital mortality rates, and intensive care unit (ICU) treatment. Finally (3) to evaluate the annual direct costs of treating vertebral fractures for the German healthcare insurance system based on a G-DRG code analysis.

## Results

1. The development of the nationwide incidence rate of vertebral fractures from 2009 to 2019.

**Figure 1:**
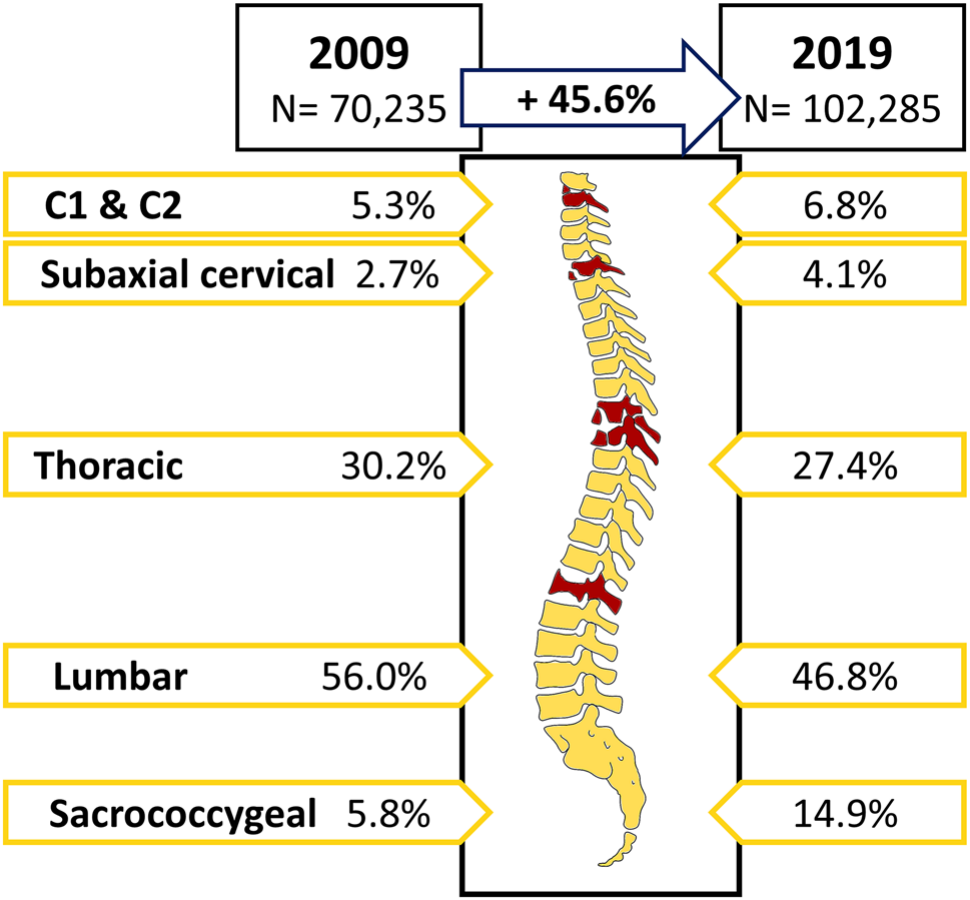
The distributions of vertebral fractures by anatomical localization comparing data from 2009 and 2019 (designed with Inkscape 1.1 and Microsoft PowerPoint).

In total 955,091 fractures of the spine were registered between 2009 and 2019, with 70,235 recorded in 2009. The total number increased by 45.6% to 102,285 fractures in 2019, giving a change in the incidence rate from 105.8/100,000 inhabitants to 150.7/100,000 inhabitants (Fig. 1). Of all vertebral fractures 57.0% were documented in female patients. (Table 1). The proportion of patients aged 70 years or older increased from 58.4% in 2009 to 68.9% in 2019.

**Table 1:** Development of vertebral fracture incidence from 2009 to 2019.

Year	Absolute number	German population 20 years or older	Change in total numbers relative to 2009 [%]	Incidence per 100,000 inhabitants per year	Incidence relative to the preceding year [%]	Ratio /female/male	Ratio aged < 70 years/ ≥ 70 years
2009	70,235	66,400,066	–	105.8	–	62/38	42/58
2010	74,588	66,549,975	+ 6.2	112.1	+ 6.0	62/38	40/60
2011	78,065	65,398,514	+ 11.2	119.4	+ 6.5	62/38	38/62
2012	79,426	65,665,069	+ 13.1	121.0	+ 1.3	62/38	36/64
2013	83,992	65,943,867	+ 19.6	127.4	+ 5.3	62/38	36/64
2014	86,865	66,677,665	+ 23.7	130.3	+ 2.3	63/37	34/66
2015	89,221	67,097,676	+ 27.0	133.0	+ 2.1	62/38	34/66
2016	94,706	67,440,230	+ 34.9	140.4	+ 5.6	63/37	33/67
2017	96,648	67,540,025	+ 37.6	143.1	+ 1.9	63/37	33/67
2018	99,060	67,724,921	+ 41.0	146.3	+ 2.2	63/67	32/68
2019	102,285	67,864,036	+ 45.6	150.7	+ 3.0	64/36	31/69

Female/male ratio and ratio aged < 70 years and ≥ 70 years. Changes were calculated relative to the incidence in 2009 und relative to the preceding year.

Figure 1 provides an overview of the distributions of vertebral fractures by anatomical localization comparing data from 2009 and 2019. Fractures of the lumbar spine were the most common vertebral fractures accounting for 56.0% in 2009 and 46.8% in 2019, followed by fractures of the thoracic spine (30.2% in 2009 and 27.4% in 2019). While sacrococcygeal fractures made up 5.8% of all vertebral fractures in 2009 their proportion increased to 14.9% in 2019.

**Figure 2:**
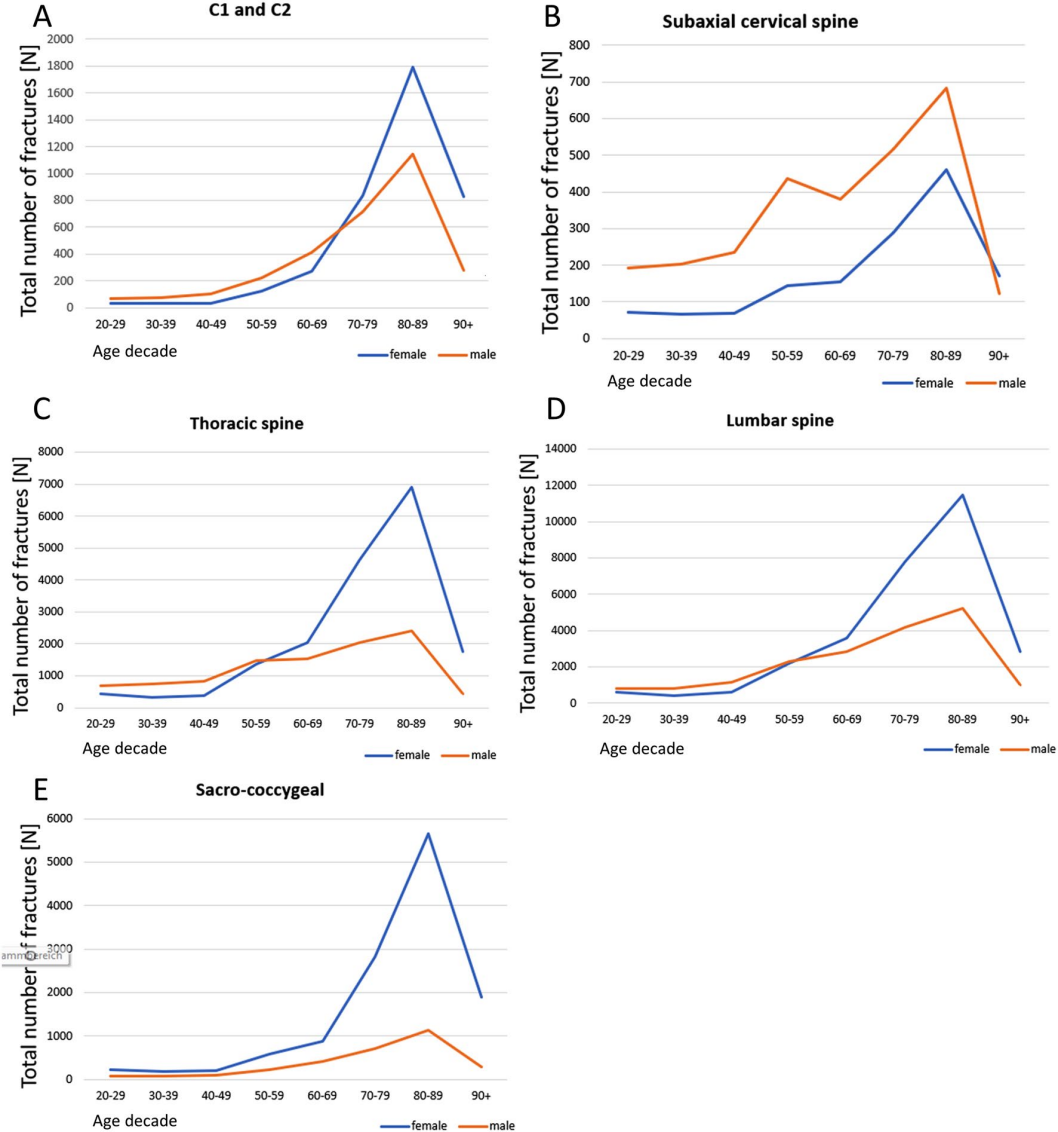
Age distribution of vertebral fractures in 2019 as a function of sex in craniocaudal order of the anatomical localization at the spine (**A**–**E**).

The most frequently fractures in 2019 were located at the lumbar spine with 47,874 registered cases and an incidence of 70.5 per 100,000 inhabitants. The most frequently documented fractures were L1 fractures, accounting for 21.9% of all cases, followed by Th11 and Th12 fractures (cumulative 16.4%) and sacrum fractures (14.3%). In 8.9% of all cases, an L1 fracture was documented as concomitant diagnosis. Lumbar spine fractures were predominantly documented in women (61.7%), with 26.4% registered in the age group 70–79 years. 38.8% of all lumbar spine fractures occurred in female patients in the age group 80–89 years, and 9.7% involving female patients aged 90 years or older (Fig. 2D). Men in the age group 80–89 years accounted for 28.4% of all lumbar spine fractures (Fig. 2D). Fractures of the thoracic spine were second most common, with 28,057 hospitalized cases in 2019 and an incidence of 41.3 per 100,000 inhabitants. The female-to-male ratio in cases with thoracic fractures was 64/36 in 2019. The highest incidences were registered in women aged 80–89 years (38.6%), 70–79 years (25.9%), and female patients aged 60–69 years (11.5%). Only 23.6% of all thoracic vertebral fractures occurred in men aged 80–89 years and 20.0% in men aged 70–79 (Fig. 2C). Fractures of the sacrum and coccyx were the third common, with an incidence of 21.7 per 100,000 inhabitants in 2019. More women than men suffered from thoracic spine fractures (female-to-male ratio 82:18), with female patients in the age group 80–89 years comprising the largest cohort (45.4%), followed by female patients aged 80–89 years (30.2%) and older than 90 years (15.3%). For male patients, most of the cases occurred in the age group 80–89 years accounting for 30.2% of all sacrococcygeal fractures (Fig. 2E). From all sacrococcygeal fractures, regardless of sex, 78.8% were documented in patients aged 70 years or older.

With 5,593 cases and an incidence of 8.2 per 100,000 inhabitants, fractures of the axis (C2) were the fourth most common spine fractures in 2019, mainly affecting female patients (57.8%). When combinend with atlas (C1) fractures they made up 6.8% of all vertebral fractures in 2019 (Fig. 1). Again, most atlas & axis fractures were seen in females (56.6%), with 67.0% occuring in patients older than 70 years. The highest number of female cases occured in the 80–89 year age group (45.4%), followed by the 70–79 year age group (21.0%) and patients older than 90 years (21.0%) (Fig. 2A). In males, the majority of cases occurred in patients aged 80–89 years (37.8%), 70–70 years (23.7%), and 60–69 years (13.6%). In 2019, fractures of subaxial cervical spine made up 4.1% of all registered spine fractures. The documentation of 4,201 subaxial spinal fracture cases, resulting in an incidence of 6.2 per 100,000 inhabitants in 2019, revealed a higher percentage of male patients (66.0%) compared to female patients (34.0%). The majority of subaxial spine fractures in male patients were seen at the age group 80–89 years (24.7%), 70–79 years (18.6%), and 50–59 years (15.7%). In females, the highest number of fractures was documented in patients aged 80–89 years (32.3%), followed by 70–79 years (20.3%), and 60–69 years (10.9%) (Fig. 2B). Focusing only on the geriatric population aged 70 years or older, substantial increases in total fracture numbers were observed between 2009 and 2019: The number of cervical, thoracic, and lumbar vertebral fractures in geriatric patients increased by 139.4%, 55.0% and 37.7%, respectively. The steepest increase in total numbers of fractures was seen at the sacrococcygeal region with an increase from 2557 to 11,955 (+ 367.5% from 2009 to 2019).

Comparing the numbers of vertebral fractures by localization from 2009 to 2019 (Table 2), the highest increase was found in fractures of the sacrum (+ 306.6%) and at the subaxial cervical spine (+ 121.2%), followed by atlas (+ 106.4%) and axis fractures (+ 82.5%). The numbers of thoracic spine fractures (+ 32.2%) and lumbar spine fractures (+ 21.7%) increased significantly. There was only a slight increase in the incidence of coccygeal fractures (+ 4.4%). A detailed analysis of the incidences and total numbers of vertebral fractures from 2009 to 2019 by anatomical localization, sex and age is provided in Supplementary Material 1A–F. The complete information on the total numbers and incidence rates of vertebral fractures by anatomical localization, as well as the relative change compared to 2009, is available in Supplementary Material 2.

(B) Secondary diagnoses, OPS codes and in-hospital mortality of vertebral fracture cases in 2019.

**Table 2:** Fracture incidence, total numbers, change in total numbers relative to 2009, female/male ratio and ratio aged < 70 years and ≥ 70 years by anatomical localization in 2019.

Anatomical localization	Absolute number	Incidence per 100,000 inhabitants	2019 relative to 2009 [%]	Ratio female/male	Ratio aged < 70 years/ ≥ 70 years
Atlas (C1)	1385	2.0	+ 106.4	52/48	24/76
Axis (C2)	5593	8.2	+ 82.5	58/42	19/81
Subaxial cervical spine	4201	6.2	+ 121.2	34/66	47/53
Thoracic spine	28,057	41.3	+ 32.2	64/36	35/65
Lumbar spine	47,874	70.5	+ 21.7	62/38	32/68
Sacrum	14,748	21.7	+ 306.6	82/18	21/79
Coccyx	427	0.6	+ 4.4	75/25	44/56

In 2019, a total of 1,016,897 secondary diagnoses were documented, averaging 9.7 per case, with 6,282 different ICD-10 codes used. In 18,773 (17.9%) cases osteoporosis (M80.- and M81.-) was documented as secondary diagnosis. Type II diabetes mellitus (E11.-) was documented in 17,006 (16.2%) cases. Secondary diagnoses of malignant neoplasms were documented in 2.5% of cases. In 61,306 (58.4%) cases a concomitant vertebral fracture was documented, most frequently located at the lumbar spine (28.9%). The most common comorbidities, along with concomitant vertebral fractures, and SCI are listed in Table 3. There were 1,148 (1.1%) cases with SCI, mainly in the cervical spine (0.5% of all fractures and 47.1% of all SCI cases).

**Table 3:** Most common comorbidities and concomitant injuries in vertebral fracture cases: total numbers and share of all cases in 2019.

	Secondary diagnosis	ICD-10 code	Total number [n]	Percentage of all cases [%]
Comorbidity	Hypertension	I10.-	55,387	52.8
	Osteoporosis	M80.-; M81.-	18,773	17.9
	Atrial fibrillation	I48.-	18,238	17.4
	Type II diabetes	E11.-	17,006	16.2
	Chronic kidney disease	N18.1-; N19	15,876	15.1
	Congestive heart failure	I50.-	13,243	12.6
	Hypokalemia	E87.6	12,892	12.3
	Coronary arterial disease	I25.0; I25.10-.19	10,610	10.1
Concomitant vertebral fractures	Atlas	S12.0	1025	1.0
	Axis	S12.1	2372	2.3
	Subaxial cervical spine	S12.21-.25; S12.7	4664	4.4
	Thoracic spine	S22.-	18,420	17.5
	Lumbar spine	S32.-	30,293	28.9
	Sacrum	S32.1	4532	4.3
SCI	Cervical spine	S14.10-.13; S14.70-.77	541	0.5
	Thoracic spine	S24.10-.12; S24.70-.77	412	0.4
	Lumbar spine	S34.10-.11; S34.70-.75	195	0.2

In 2019 1833 different OPS codes were used, resulting in the documentation of 493,043 OPS codes at total, and on average of 4.8 codes per case. The analysis of OPS codes of all documented cases showed a total number of approaches to the spine of 27,314 (26.1% of cases; Table 4). Instrumentation/fixation procedures (5-83b.-) were documented in 21.3%. Kyphoplasty (5-839.a0-3) was conducted in 15.2% of cases, and vertebroplasty was documented in 1.6% of cases. In 4.7% of cases, screws were augmented. Autografts were used more often (2.8%) than allografts (1.7%). In 60.5% of cases, a native computed tomography (CT) of the spine (3-203; 47.7%) or the pelvis (3-206; 12.8%) was applied. Native Magnet-resonance imaging (MRI; 3-802) of the spine was used in 28.8% of cases.

**Table 4:** Frequencies and percentages of documented procedures according the OPS of vertebral fracture cases in 2019.

Procedures	OPS code	Total number [n]	Percentage of all cases [%]
Approach to the cervical spine	5-030.-	4890	4.7
Approach to the thoracic spine	5-031.-	8671	8.3
Approach to the lumbar spine	5-032.-	13,753	13.1
Instrumentation/Fixation	5-83b.-	22,330	21.3
Kyphoplasty	5-839.a0-3	15,904	15.2
Fusion surgery/Spondylodesis	5-836.-	6479	6.2
Screw augmentation	5-83w.0	4882	4.7
Implant removal	5-839.0	4165	4.0
Use of autograft	5-835.9	2947	2.8
Vertebral canal decompression	5-033.0; 5-839.60-63	2260	2.7
Vertebral body replacement	5-837.-	2343	2.2
Use of allograft	5-835.a-.x	1801	1.7
Vertebroplasty	5-839.9-5	1714	1.6

The overall in-hospital mortality rate associated with vertebral fractures was 2.0% in 2019. The highest mortality rate (6.7%) was seen in cases with atlas & axis fractures. In cases with atlas & axis fractures ICU treatment was most common (27.9%), while overall, 10.1% of cases received ICU treatment. Detailed numbers of in-hospital deaths and ICU treatment are displayed in Table 5.

(C) The direct healthcare costs of vertebral fractures for inpatient cases based on a G-DRG analysis in 2019.

**Table 5:** Percentages of in-hospital deaths and cases with ICU treatment depending on the anatomical localization of the fracture in 2019.

	Atlas & axis		Subaxial cervical spine		Thoracic spine		Lumbar spine		Sacrum	
	n	%	n	%	n	%	n	%	n	%
Number of in-hospital deaths	475	6.7	207	4.7	486	1.6	655	1.3	253	1.7
Cases with ICU treatment	1972	27.9	198	4.5	3157	10.7	4026	8.2	1243	8.3

On average, the hospital stay for all patients with vertebral fractures was 10.7 ± 9.6 days. The longest mean hospital stay per case (13.9 ± 9.8 days) was documented cases with sacrum fractures. On average in 16.8% of all cases high clinical complexity levels (PCCL ≥ 3) were seen. In patients with atlas & axis fractures 24.4% were classified as PCCL ≥ 3, in patients with subaxial cervical fractures 24.7%, and in patients with fractures of the sacrum 28.8%. Table 6 provides an overview of the mean hospital stay and the proportion of cases according to the PCCL scoring.

**Table 6:** Mean hospital stay and distribution of the PCCL scoring depending on the anatomical localization of vertebral fractures in 2019.

	All vertebral fractures	Atlas & axis	Subaxial cervical spine	Thoracic spine	Lumbar spine	Sacrum
Mean hospital stay [d]	10.7	10.8	10.6	10.1	10.3	13.0
Standard deviation	9.6	11.2	13.4	9.3	9.0	9.8
PCCL						
0	58.4%	50.3%	47.1%	61.4%	67.0%	31.6%
1	14.1%	13.7%	16.7%	13.0%	12.1%	22.4%
2	10.6%	11.5%	11.6%	9.9%	8.8%	17.2%
3	10.4%	13.7%	12.8%	9.3%	7.9%	18.2%
4	5.1%	7.8%	8.7%	4.9%	3.5%	8.5%
5	1.2%	2.5%	2.9%	1.3%	0.7%	1.9%
6	0.1%	0.4%	0.3%	0.1%	0.1%	0.2%

The most frequently used G-DRG code for vertebral fracture cases in 2019 was I68D (“Diseases and injuries of the spine not treated surgically”), used in 33.3% of cases. The second most common was I41Z (“Geriatric early rehabilitation and complex treatment for diseases and disorders of the musculoskeletal system and connective tissue”) in 10.3% of cases, followed by I09I (“Specific surgeries on the spine without complicating factors”), used in 8.6% of the cases. A detailed list of the 50 most commonly used DRG codes (in 98.9% of cases, only codes used in at least 0.05% of cases were considered), costs per case, and share of overall costs is provided in the supplementary material (Supplementary material 3).

The cost per case for the analyzed G-DRG codes ranged from €719 ± 262 (I68E: “Diseases and injuries in the spine not treated surgically, one occupancy day”) to €67,782 ± 16,714 (A09B: Ventilation > 499 h or > 249 h with intensive complex treatment). The total costs of treating inpatients in 2019 were €589,205,715, resulting in a mean cost of €5612 per case. The DRG code accounting for the largest share of overall costs was I09F: Mean costs per case: € 10,160.00 ± 3169.00; 13.4% of overall costs. Second, I41Z: Mean costs per case: € 5878.00 ± 1317.00; 10.8% of overall costs. Third ranked I68D: Mean costs per case: € 1708.00 ± 636.00; 10.2% of overall costs. 40.7% of the total costs are attributable to G-DRG codes used in each < 3.0% of all cases (n = 41 different G-DRG codes, supplementary material 3). Table 7 lists the cost per case and the share of total costs of the G-DRG codes in 2019 without cases accounting for < 3.0% of cases. When cases with codes for non-surgical treatment of vertebral disorders and injuries (I68A-E) were summed, there were 51,724 cases (49.3%), which accounted for €108,066,426 or 18.3% of the overall costs. The entirety of cases where codes for specific surgeries of the spine (I09A-I) were applied consisted of 27,381 (26.1%) cases and accounted for 44.4% of the overall costs for the in-hospital treatment of vertebral fractures in 2019 (€261,532,171).

**Table 7:** Overview of the codes that each represent more than 3.0% of the cases.

G-DRG code	Original German description	English summary	Cases [n]	Share of cases (%)	Mean cost per case [€]	Standard [€] deviation	Overall direct costs [€]	Share of overall costs (%)
I68D	Nicht operativ behandelte Erkrankungen und Verletzungen WS, mehr als ein Belegungstag oder andere Femurfraktur, außer bei Diszitis oder infektiöser Spondylopathie, ohne Kreuzbeinfraktur, ohne best. mäßig aufw., aufw. od. hochaufw. Beh.	Non-surgically treated disorders and injuries of the spine, more than one day of occupancy or other femur fracture, except for discitis infectious spondylopathy, without sacral fracture, without specific moderately complex, complex or highly complex treatments.	35,000	33.3	1708	636	59,780,000	10.1
I41Z	Geriatrische frührehabilitative Komplexbehandlung bei Krankheiten und Störungen an Muskel-Skelett-System und Bindegewebe.	Geriatric early rehabilitation complex treatment for disorders and disorders of the musculoskeletal system and connective tissue.	10,825	10.3	5878	1317	63,629,350	10.8
I09I	Bestimmte Eingriffe an der Wirbelsäule ohne komplizierende Faktoren.	Specific surgeries of the spine without complicating factors.	9050	8.6	5180	1642	46,879,000	8.0
I09F	Best. Eingriffe WS und best. kompl. Faktoren od. best. andere Eingriffe WS mit best. anderen kompl. Faktoren od. Alter kl. 16 J., oh. Eingriffe ZNS, oh. transpleuraler Zugang BWS, oh. best. langstreckige Spondylodese/Osteosynthese, oh. Diszitis.	Specific surgeries of the spine and specific complicating factors or specific other spinal procedures with specific other complicating factors or age less than 16 years, without central nervous system procedures, without transpleural access to the thoracic spine, without specific long spondylodesis/osteosynthesis, without discitis.	7785	7.4	10,160	3169	79,095,600	13.4
I68C	Nicht operativ behandelte Erkrankungen und Verletzungen WS, > 1 BT od. and. Femurfraktur, bei Para- / Tetraplegie od. mit äuß. schw. CC od. schw. CC od, Alter > 65 J., oh. kompl. Diagn. od. Kreuzbeinfraktur od. best. mäßig aufw., aufw. od. hochaufw. Beh.	Non-surgically treated disorders and injuries of the spine, > 1 day of occupancy or other femur fracture, in case of para- / tetraplegia or with extremely severe CC or severe CC or, age > 65 y., without complicating diagnosis or sacral fracture or specific moderately complex, complex or highly complex treatment.	7190	6.8	3297	1604	23,705,430	4.0
I68E	Nicht operativ behandelte Erkrankungen und Verletzungen im Wirbelsäulenbereich, ein Belegungstag	Disorders and injuries in the spine not treated non-surgically, one occupancy day	5069	4.8	719	262	3,644,611	0.6
I68B	Nicht operativ behandelte Erkrankungen und Verletzungen im Wirbelsäulenbereich, mehr als 1 BT, mit äuß. schw. oder schw. CC od. bei Para- / Tetraplegie, mit kompl. Diagn. oder ohne äuß. schw. oder schw. CC, ohne Para- / Tetraplegie bei Diszitis.	Non-surgically treated disorders and injuries in the spinal region, > 1 day of occupancy, with extremely severe or severe CC or with para- / tetraplegia, with complicating diagnoses or without extremely severe or severe CC, without para- / tetraplegia with discectomy.	4465	4.3	4689	2334	20,936,385	3.6
I09E	Bestimmte Eingriffe an der Wirbelsäule und best. komplizierende Faktoren oder best. Eingriffe an der WS mit best. anderen kompl. Faktoren und Eingriffe ZNS oder transpleuraler Zugang BWS oder best. langstreckige Spondylodese/Osteosynthese oder Diszitis.	Specific surgeries of the spine and specific complicating factors or specific surgeries of the spine with specific other complicating factors and operations of the central nerve system or transpleural access to the thoracic spine or specific long spinal fusion / osteosynthesis or discitis.	3925	3.7	13,194	3892	51,786,450	8.8
Other (each < 3.0% od overall cases)			20,464	19.5	18,441		239,748,889	40.7

Sorting according to frequency of use in 2019.

## Discussion

In this cross-sectional study, we analyzed the development of vertebral fracture incidence as a function of age, sex, and by anatomical localization, based on registry data consisting of ICD-10 diagnosis codes from all German medical institutions over eleven years. Nationwide data on comorbidities, concomitant injuries, procedures (based on OPS codes), and the G-DRG distribution were assessed based on the InEK data and report browsers for the year 2019. Whereas studies relying on data from single hospitals may yield skewed results, the findings presented here are based on national reports from the largest country of the European Union.

In 2019, a total of 102,285 fractures were registered depicting an increase of 45.6% since 2009. The incidence of all vertebral fractures was 150.7 per 100,00 inhabitants. We recently showed that fractures of the thoracic and of the lumbar spine are among the ten most common fractures in Germany in 2019^14^. We demonstrated that the majority of fractures can be found in the elderly population: The proportion of all patients aged 70 years or older was 69.0% in 2019. Further, more females than males suffered from vertebral fractures (ratio female:male = 0.6). Consistent with the literature the most common localizations of fractures were the lumbar and thoracic spine with L1 being the most commonly affected vertebral body in 21.9% of all cases^15,16^. Recently, a systematic analysis of the global burden of disease study 2019 demonstrated a steep increase of cases of 38% between 1990 and 2019 with falls being the leading course of vertebral fractures at all ages^17^. Contrary to our findings the Global Burden of Disease study 2019 revealed an age-standardized rate of incidence of vertebral fractures of 92.2/100,000 in females and 125.3/100,000 in males^7^. This difference in the epidemiological distribution of vertebral fractures may be attributed to various factors such as age distribution, lifestyle patterns, and cultural and environmental influences, implying that the role of osteoporotic vertebral fractures may not be as pronounced worldwide as in Germany. Similar to our findings Blecher et al. reported an overall increase of vertebral fractures of 64.0% between 2003 and 2017 in a longitudinal trend analysis of discharged patient state database in Washington State^18^. They underlined the uptrend in the vertebral fractures of the elderly and found the highest increase in cervical spine fractures (+ 123.0%)^18^. Den Ouden et al. reported on an increasing number of vertebral fractures in a level 1 trauma center in a 10-year period^19^. Consistent with our results they found a larger increase in the number of spine fractures in patients over 65 years of age compared with younger patients. In their cohort of exclusively traumatic vertebral fracture patients, 40.8% were female and 59.2% were male, with a mean age of 52.0 years and most fractures occurred at the lumbar spine^19^. In a nationwide analysis of traumatic vertebral fractures in the Netherlands from 2010 to 2017 Smits et al. showed an incidence of vertebral fractures of 24.0 per 100,000 inhabitants in 2017^2^. The currently presented data consist of the totality of documented hospitalized cases with vertebral fractures, including elderly patients and cases suffering minor trauma. The age and sex distribution of the current study population, with elderly women accounting for a large part of the cases underlines this fact. The essential role of insufficiency fractures can be most obviously seen in the increase of the incidence of sacral fractures by 307.0% compared to 2009. Vertebral fractures are a hallmark of osteoporosis. They represent the most frequent osteoporotic fracture^20^. The aging EU population, particularly those over 80 years is expected to contribute to an increase of osteoporosis and associated fractures: The number of prevalent vertebral fractures is expected to rise from 23.7 million in 2000 to 37.3 million by 2050^12^. Global deaths and disability-adjusted life-years (DALY) number attributable to low bone mineral density (LBMD) increased from 207,367 and 8,588,936 in 1990 to 437,884 and 16,647,466 in 2019, with a raise of 111.16% and 93.82%, as shown by an analysis of the global burden of disease study 2019^21^. Germany ranks number five of the countries with the worldwide highest disease burden of DALYs number in LBMD-related fractures^21^. However, it must be assumed that the sharp increase in the total number of vertebral fractures is not only due to the aging population structure in Germany. The improved availability and quality of CT and MRI must be considered to contribute to the increased documentation of vertebral and sacral fractures^22–24^. Recently, Graul et al. demonstrated the sensitivities for sacral insufficiency fractures to be 14% in X-ray, 88% in CT, and 100% in MRI^25^. They further figured out that additional pathologies were identified in MRI of the lumbar spine in 51% and pelvis in 18%, respectively^25^. They consequently advocated for extensive MRI diagnostic of the lumbar spine including the sacrum for symptomatic elderly patients^25^. In the current population in 60.5% a CT and in around 30% an MRI of the spine or the pelvis were applied in 2019.

The most commonly documented comorbidities in the studied population were hypertension, osteoporosis, atrial fibrillation, type II diabetes, and chronic kidney disease. These can be assumed to be indicative for the elderly German population^26,27^. Of note, osteoporosis was documented as a secondary diagnosis in 18,773 (17.9%) cases, but the study did not screen for “M80.-” and “M81.-” as main diagnoses. In Europe, a prevalence of 18–26% of osteoporotic vertebral fractures is reported, with women being affected more often in Germany^28,29^. In their recent systematic review, Spiegl et al. point out the role of osteoporotic fractures of the spine and the importance of tailored diagnostic and therapeutic strategies^30^. The use of kyphoplasty or vertebroplasty in around 17% of cases and the use of screw augmentation in around 5.0% of cases in the current study population underline the important role of osteoporotic vertebral fractures and indicate the challenges in surgical management.

Interestingly, a high percentage of cases had concomitant vertebral fractures documented, with fractures at the lumbar spine in around 29% and at the thoracic spine in around 18%. Hypothetically these fractures partly can be accounted for poor bone quality in patients with osteoporosis: The incidence of concomitant vertebral fractures at first contact in patients with osteoporotic vertebral fractures has been reported to be 26% in a register-based epidemiological study^31^. Further, the occurrence of multilevel contiguous osteoporotic lumbar compression fractures has been shown before^32^. Also in non-osteoporotic patients multilevel vertebral injuries are common, with incidences around 20–30%^33–35^. Especially occult, non-continuous vertebral injuries bare the risk of delayed diagnosis. Therefore some authors recommend considering whole spine MRI in vertebral trauma patients^33,35^.

We found that 1.1% of cases of vertebral fractures were associated with SCI, which is comparativley low compared to other studies. Den Ouden et al. reported an SCI rate of 8.5% in their population of traumatic vertebral fracture patients in 2016^19^. In their nationwide analysis of traumatic vertebral fractures, Smiths et al. showed an SCI rate of 5.5%, mainly associated with cervical spine fractures^2^. A reason for the comparatively low rate of SCIs in our study could be the heterogenous population, which included low-energy trauma mechanisms and elderly patients. It can be hypothesized, that the association with SCI partly accounts for the severity of cervical spine trauma cases, which is reflected in the high percentage of ICU treatment of 27.9% for atlas and axis fractures and of 4.5% for subaxial cervical spine fractures.

Based on the most used G-DRG codes, we estimated the overall cost of hospitalized patients with vertebral fractures in Germany to be around €590 million in 2019, with an average costs per case of €5612. This represents a cross-section of the different case complexities ranging from €719 to €67,782 depending on the G-DRG codes. The majority of cases was coded as I68D, for non-surgical treatment of vertebral fractures, accounting for 33% of cases. I09F coding for specific surgical vertebral procedures with complicating factors only made up 7.4% of cases but accounted for 13.4% of the overall costs. Cases with codes for non-surgical treatment of vertebral fractures accounted for 18.3% of the overall costs, representing 49.3% of all cases. In contrast, cases with codes for specific surgical treatment made up 44.4% of the overall costs, while only representing 26.1% of the cases. Cases with cervical spine fractures and fractures of the sacrum had the highest shares of high clinical complexity levels (PCCL ≥ 3).

The data on up-to-date nationwide cost analysis for vertebral fractures is sparse. An analysis of approximately 200,000 hospitalizations in the U.S. demonstrated that the total national charges associated with cervical spine fractures with and without SCIs combined exceeded $1.3 billion US in 2006^36^. For the EU the total hospital costs for vertebral fractures have been estimated at €377 Million per year early in 2003^37^. Based on an analysis of 491 patients treated conservatively for vertebral fractures between 1999 and 2008 Aras et al. estimated the total health cost at €18,919, €8571, and €5526 for cervical, thoracic, and lumbar regions, respectively^38^. In 2004 van der Roer estimated the average costs for stable fractures without neurological deficits treated nonoperatively at €5100, for unstable fractures without neurological deficits, treated either nonoperatively, at €12,500 or operatively at €19,700^39^. The costs for unstable fractures with neurological deficits were estimated at €31,900^39^. These estimations are in the range of the average cost for the respective G-DRG on which our analysis is based.

The increase in fracture incidence poses a challenge for stakeholders in the healthcare systems. Fragility fractures were projected to be 928,000 in 2025 with an estimated socioeconomic burden of €11,261 million^20^. Vertebral fractures, even treated conservatively, incur substantial healthcare utilization and costs. However, indirect health care costs such as the costs of work absenteeism and disability add to the overall costs, and could not be accessed in this study. Further studies on the cost-effectiveness, cost-utility, and long-term costs of vertebral fractures are necessary to comprehend the total economic burden. The current analysis summarizes the up-to-date costs of vertebral fractures in the German population in 2019 and therefore provides important information for the planning of preventive and treatment strategies.

## Strengths and limitations

The main strength of the current study is the nationwide analysis over one decade. To provide comprehensive information on different aspects of the inpatient treatment of vertebral fractures two data sources were employed: Data based on the Destatis analysis (2009–2019) and data based on the InEK data and report browser (2019). Correct coding of diagnoses can be assumed, given that DRG lump sum payment relies on it and is strictly controlled by the Medical Service of Health Funds. However, the study is limited by the fact that the trauma mechanisms could not be determined, and only inpatient data were available, potentially underestimating the reported fracture incidences. Furthermore, the databases did not enable to evaluate the age of the fracture, although it can be assumed that most hospitalized cases suffered a fresh fracture. Moreover, a detailed analysis was conducted only for primary diagnoses. We showed, that in this population in 58.4% of cases, an additional vertebral fracture was documented as a concomitant diagnosis. The overall number of vertebral fractures might be underestimated because concomitant diagnoses were not screened for all relevant primary diseases.

In conclusion, the findings of this nationwide cross-sectional study over one decade show a steep increase in the incidence rate of vertebral fractures, highlighting the challenge for stakeholders in the healthcare systems. The associated costs of approximately €590 million in 2019 emphasize the importance of effective management strategies. The age and sex distribution suggest a growing incidence of osteoporotic vertebral fractures, which underlines the need for prevention and guideline-based therapy as well as comprehensive geriatric assessment. The high precentage of complex cases requiring surgical and ICU treatment supports the need for expanding the nationwide implementation of spinal care centers.

## Methods

### Federal Statistical Office of Germany (Destatis)

Data consisting of annual ICD-10 diagnosis codes from German medical institutions between 2009 through 2019 was provided by the Federal Statistical Office of Germany (Destatis). The total number of vertebral fractures was quantified using the ICD-10 Codes “S12.0”, “S12.1”, “S12.2-”, “S22.0-”, “S32.0-”, “S32.1” and “S32.2” (Table 8) and analyzed as a function of anatomical localization, sex and age in 10-year increments for patients older than 20 years from 2009 through 2019, respectively. Incidence rates were calculated based on Germany’s historical population aged 20 years or older provided by Destatis^40^. Here, the number of inhabitants in each of the 16 German federal states was considered by year of birth for each year of the period 2009 through 2019. The deadline for each year was December 31.

**Table 8:** Used ICD-10 codes for vertebral fractures with descriptions.

ICD-10 code	Description
S12.0	Atlas (C1)
S12.1	Axis (C2)
S12.2-	Subaxial cervical spine (C3–C7)
S22.0-	Thoracic spine (Th1–Th12)
S32.0-	Lumbar spine (L1-L5)
S32.1	Sacrum (S1-S5)
S32.2	Coccyx

### Institute for the hospital remuneration system (InEK GmbH)

Per section 17b of the German Hospital Financing Act (KHG), a universal, performance-based, and flat-rate remuneration system has been introduced for general hospital services. The basis for this is the G-DRG system (German Diagnosis Related Groups system), whereby each inpatient case of treatment is remunerated employing a corresponding DRG lump sum payment.

The InEK GmbH provides detailed data on the main diagnoses (ICD-10 coded), encoding of secondary diagnosis for comorbidities and concomitant diagnoses (ICD-10 coded), procedures (OPS coded), and the G-DRG distribution. Data is accessible via the InEK Data Browser^41^. The Browser enables analysis back to the year 2019. The following comprehensive analysis was made only for the year 2019: Based on the ICD-10 codes for vertebral fractures, displayed in Table 8 data for total case numbers, numbers of in-hospital deaths and numbers of cases treated in an ICU were extracted. Further, the data on comorbidities, concomitant diagnoses, OPS codes, time of hospital stay (mean ± standard deviation), and G-DRG codes were analyzed. A specific G-DRG code corresponds to each case that was determined based on the ICD code. Cases that were treated for the same diagnosis within the same year were grouped. Thus, no duplicates were recorded.

To estimate the direct costs for inpatient treatment of vertebral fractures the G-DRG Report browser was employed^42^. The InEK Report browser allows for the calculation of costs based on various factors such as length of hospital stay, the severity of illness, and treatment procedures. This data determines appropriate reimbursement rates for hospitals and ensures fair and consistent payment for medical services. The G-DRG Report Browser 2019 displays the calculation results for the G-DRG system 2019 on the DRG level. The share of cases according to the Patient Clinical Complexity Level (PCCL) was adopted from the InEK Data Browser. The PCCL value is calculated in a complex procedure from the concomitant diagnosis values (complication or comorbidity level values—CCL) and indicates the severity of the complication or comorbidity based on results between 0 (no CC) and 6 (most severe CC). Costs according to the distribution of applied G-DRG codes, that were used in at least 0.05% of cases were added up proportionally and calculated as the mean value per case.

### Ethics declarations

Methods were carried out in accordance with local data privacy guidelines and ethical regulations.

## Supplementary Information

Supplementary Information 1.

Supplementary Information 2.

Supplementary Information 3.

Supplementary Legends.

## Data Availability

The datasets analyzed during the current study are available from the corresponding author upon reasonable request. The raw data is publicly available and provided by Destatis and the InEK data browser.
