# Supplementary figures and images for: Increased incidence of vertebral fractures in German adults from 2009 to 2019 and the analysis of secondary diagnoses, treatment, costs, and in-hospital mortality
Source: Sci Rep. 2023 Apr 28;13:6984. doi: 10.1038/s41598-023-31654-0 (PMC10147602; doi:10.1038/s41598-023-31654-0)

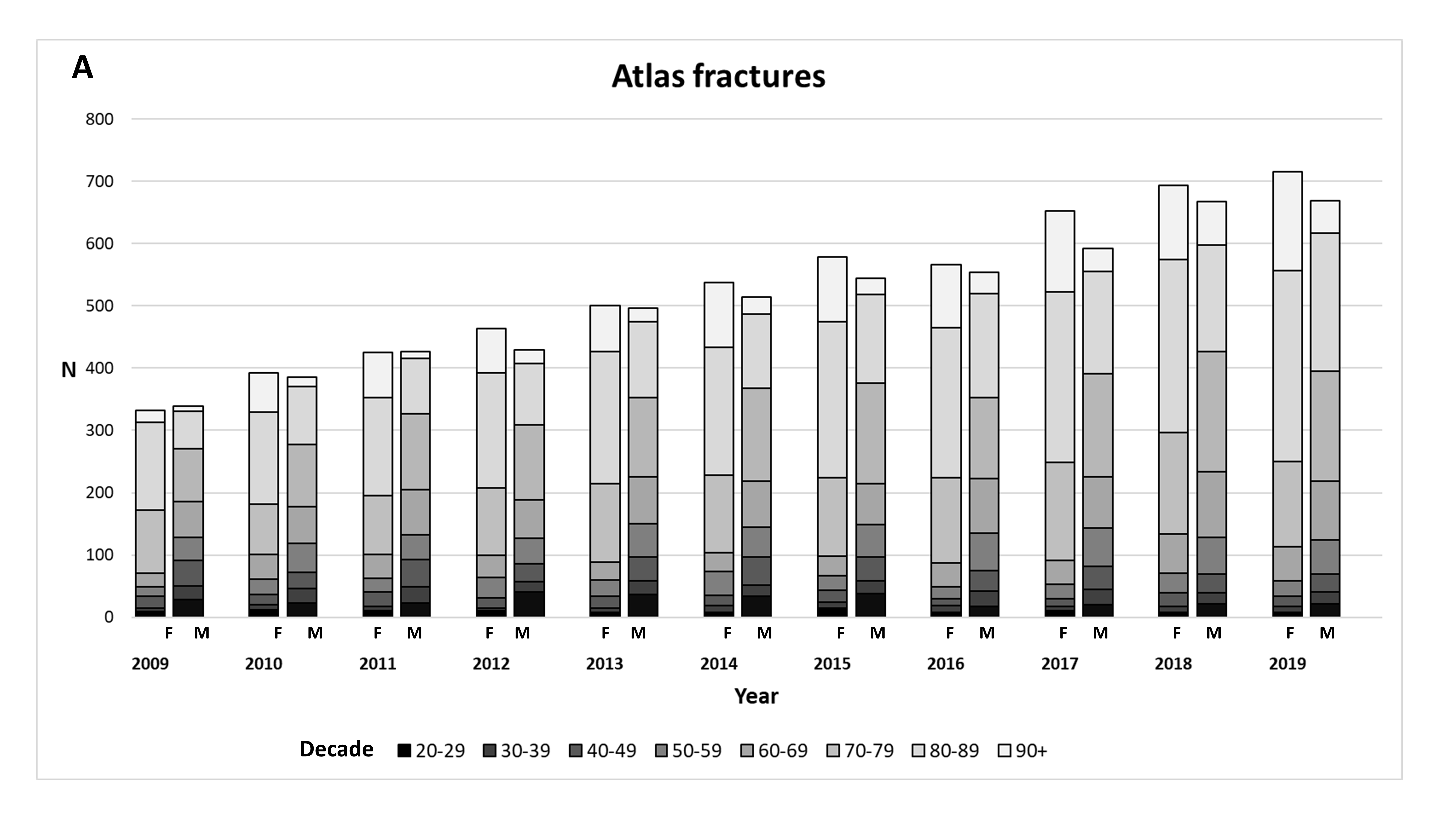

Supplement: Supplementary file 1 — Supplementary Information 1. [file 41598_2023_31654_MOESM1_ESM.zip › FIG/41598_2023_31654_MOESM1_ESM.TIF]

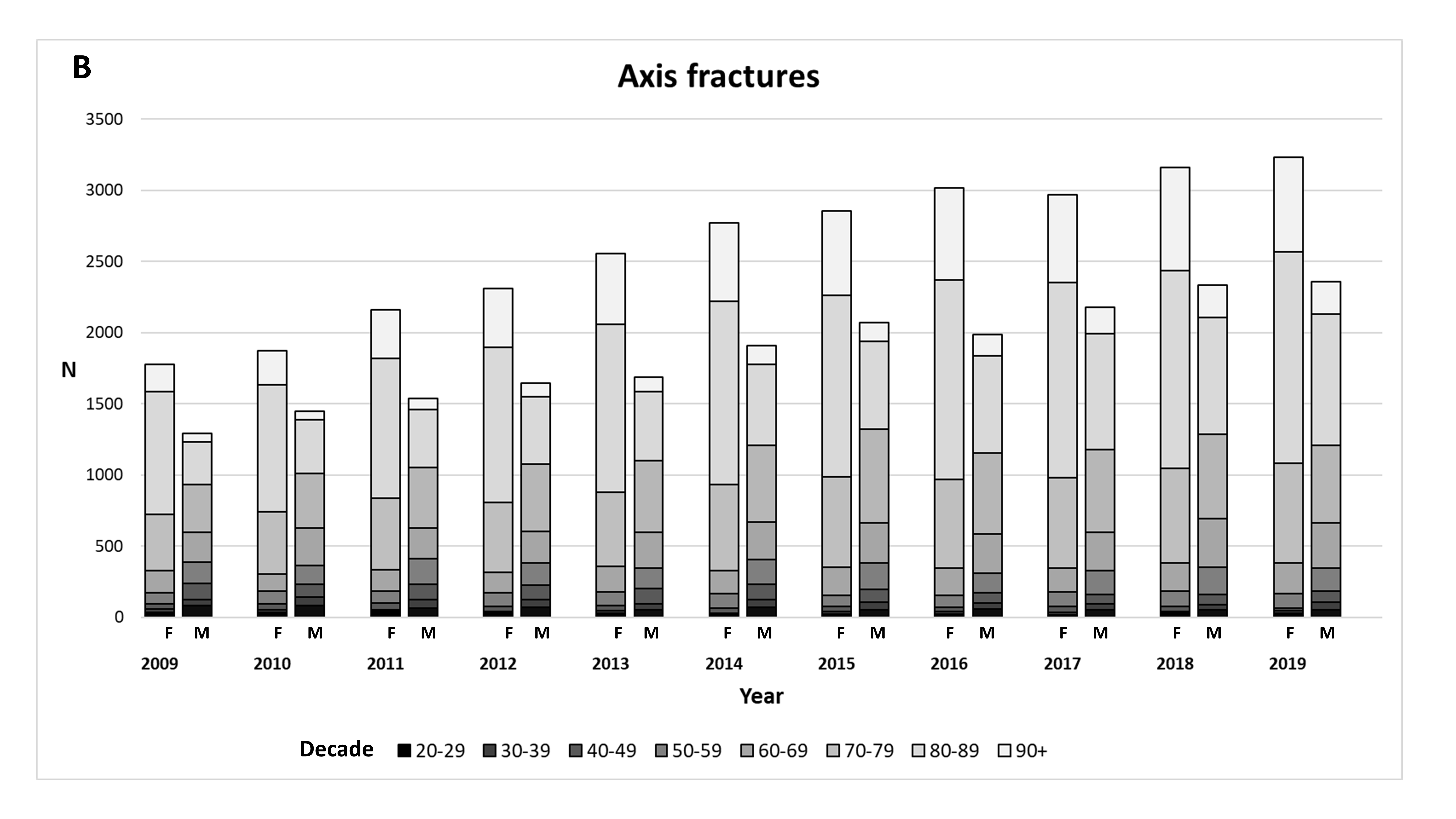

Supplement: Supplementary file 1 — Supplementary Information 1. [file 41598_2023_31654_MOESM1_ESM.zip › FIG/Supp 1B.TIF]

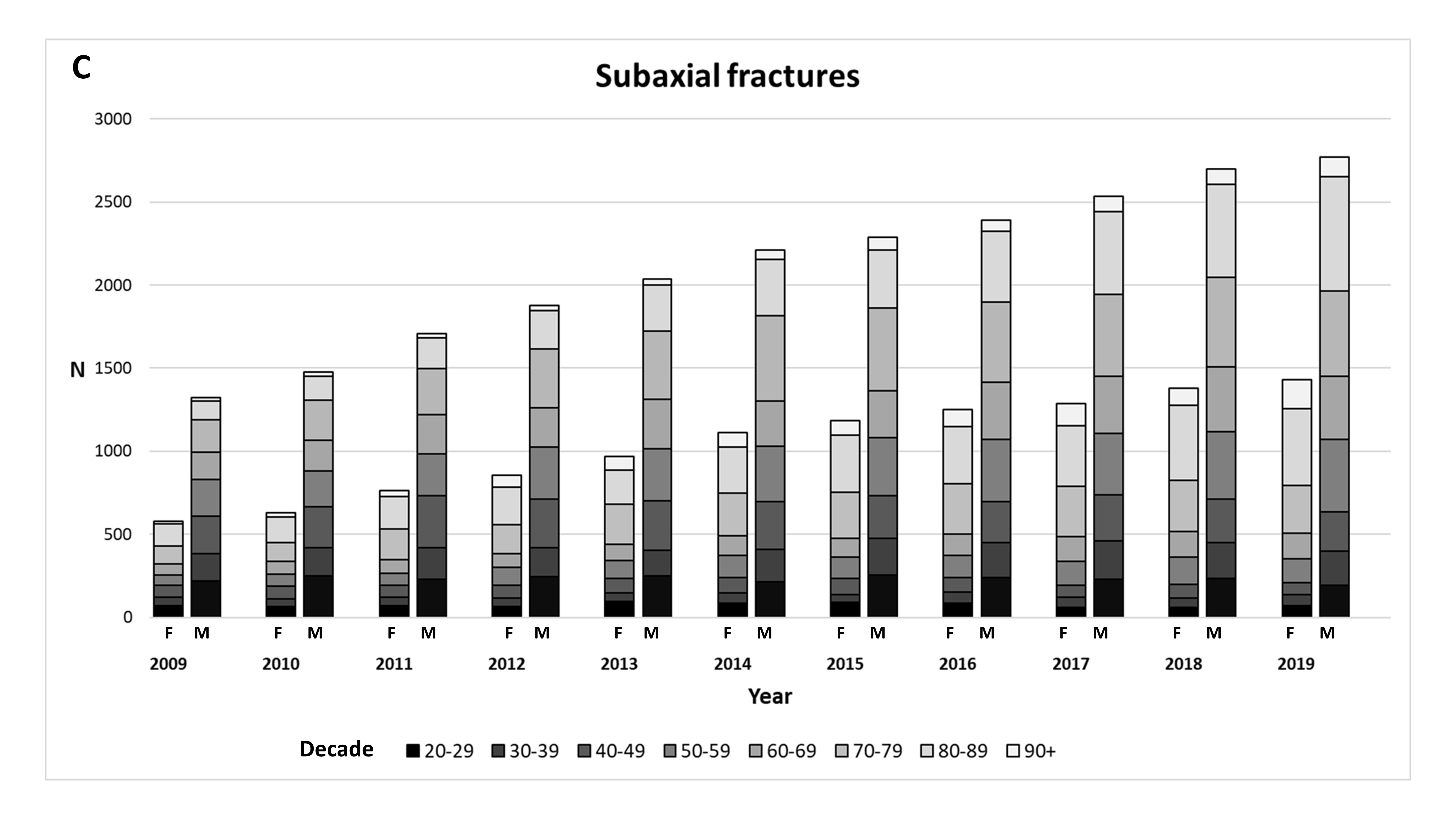

Supplement: Supplementary file 1 — Supplementary Information 1. [file 41598_2023_31654_MOESM1_ESM.zip › FIG/Supp 1C.TIF]

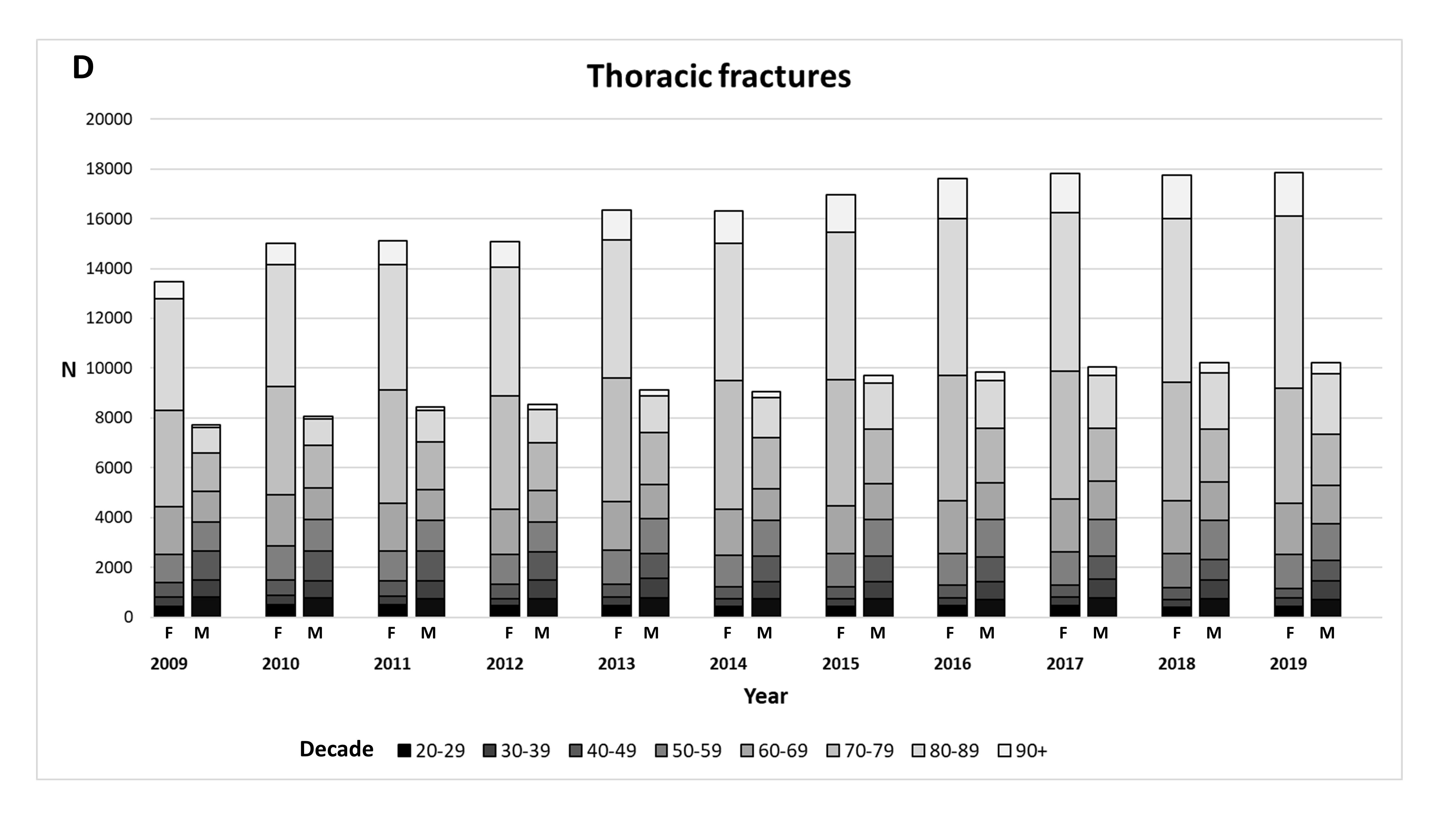

Supplement: Supplementary file 1 — Supplementary Information 1. [file 41598_2023_31654_MOESM1_ESM.zip › FIG/Supp 1D.TIF]

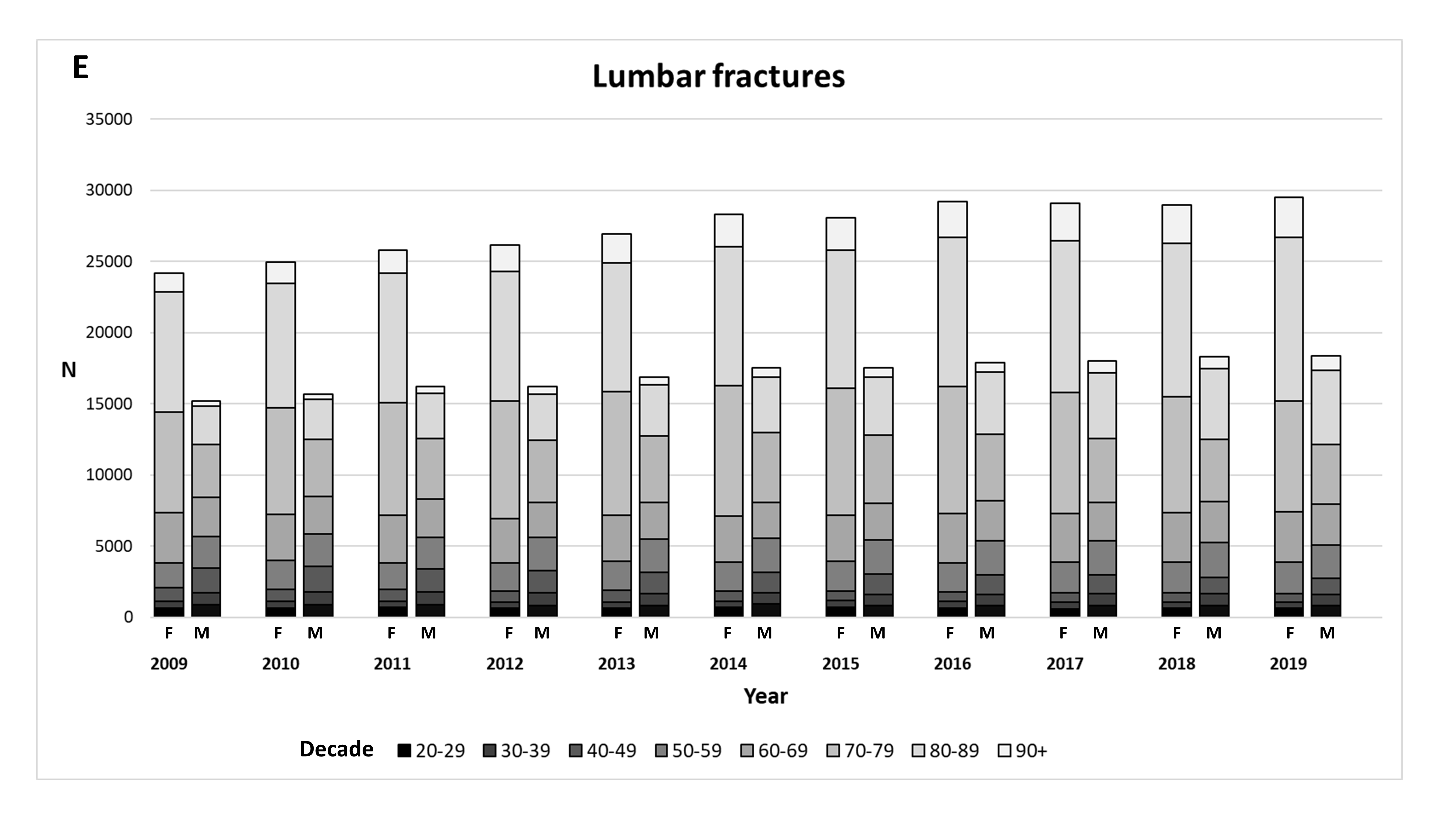

Supplement: Supplementary file 1 — Supplementary Information 1. [file 41598_2023_31654_MOESM1_ESM.zip › FIG/Supp 1E.TIF]

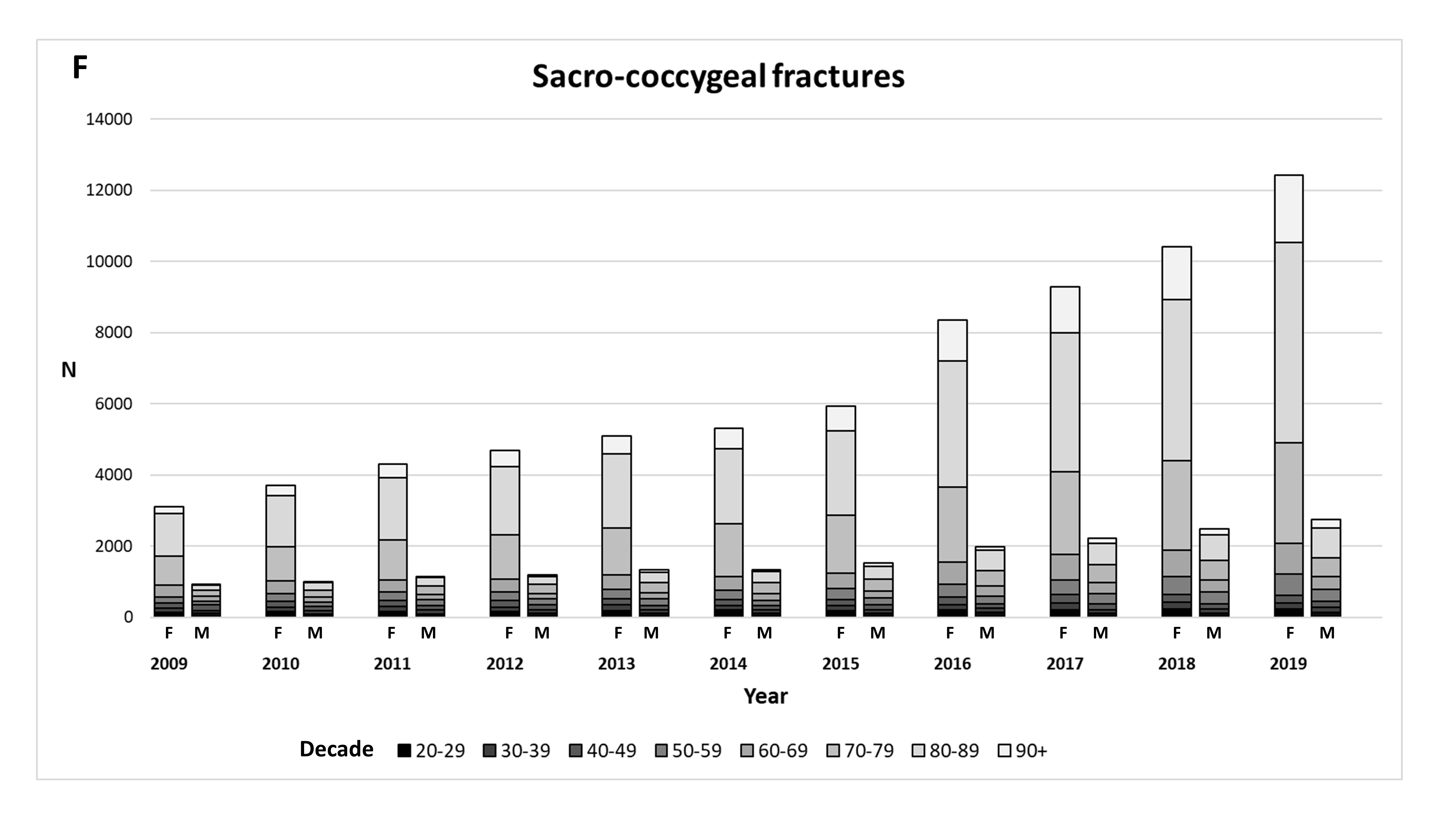

Supplement: Supplementary file 1 — Supplementary Information 1. [file 41598_2023_31654_MOESM1_ESM.zip › FIG/Supp 1F.TIF]
