# Supplementary material for: Increased incidence of vertebral fractures in German adults from 2009 to 2019 and the analysis of secondary diagnoses, treatment, costs, and in-hospital mortality
Source: Sci Rep. 2023 Apr 28;13:6984. doi: 10.1038/s41598-023-31654-0 (PMC10147602; doi:10.1038/s41598-023-31654-0)
Supplement: Supplementary file 2 — Supplementary Information 2. [file 41598_2023_31654_MOESM2_ESM.docx]

|  | | **Atlas** | | | **Axis** | | | **Subaxial cervical spine** | | | **Thoracic spine** | | | **Lumbar spine** | | | **Sacro-coccygeal** | | |  |
| --- | --- | --- | --- | --- | --- | --- | --- | --- | --- | --- | --- | --- | --- | --- | --- | --- | --- | --- | --- | --- |
|  |  |  |  |  |  |  |  |  |  |  |  |  |  |  |  |  |  |  |  |  |
| Year | German  population (Mio.) | Total numbers | 2019 relative to 2009 [%] | Prevalence per 100,000 inhabitants | Total numbers | 2019 relative to 2009 [%] | Prevalence per 100,000 inhabitants | Total numbers | 2019 relative to 2009 [%] | Prevalence per 100,000 inhabitants | Total numbers | 2019 relative to 2009 [%] | Prevalence per 100,000 inhabitants | Total numbers | 2019 relative to 2009 [%] | Prevalence per 100,000 inhabitants | Total numbers | 2019 relative to 2009 [%] | Prevalence per 100,000 inhabitants |  |
| 2009 | 66.4 | 671 | 0.0 | 1.0 | 3,065 | 0.0 | 4.6 | 1,899 | 0.0 | 4.1 | 21,220 | 0.0 | 32.0 | 39,344 | 0.0 | 59.3 | 4,036 | 0.0 | 6.1 |  |
| 2010 | 66.5 | 778 | 15.9 | 1.2 | 3,320 | 8.3 | 5.0 | 2,103 | 10.7 | 4.2 | 23,088 | 2.6 | 34.7 | 40,596 | 3.2 | 61.0 | 4,703 | 16.5 | 7.1 |  |
| 2011 | 65.4 | 852 | 27.0 | 1.3 | 3,699 | 20.7 | 5.7 | 2,468 | 30.0 | 4.3 | 23,564 | 4.5 | 36.0 | 42,023 | 6.8 | 64.3 | 5,459 | 35.3 | 8.3 |  |
| 2012 | 65.7 | 893 | 33.1 | 1.4 | 3,955 | 29.0 | 6.0 | 2,733 | 43.9 | 4.2 | 23,643 | 2.1 | 36.0 | 42,332 | 7.6 | 64.5 | 5,870 | 45.4 | 8.9 |  |
| 2013 | 65.9 | 997 | 48.6 | 1.5 | 4,247 | 38.6 | 6.4 | 3,002 | 58.1 | 4.3 | 25,490 | 5.0 | 38.7 | 43,828 | 11.4 | 66.5 | 6,428 | 59.3 | 9.7 |  |
| 2014 | 66.7 | 1,051 | 56.6 | 1.6 | 4,674 | 52.5 | 7.0 | 3,323 | 75.0 | 4.4 | 25,361 | 7.1 | 38.0 | 45,801 | 16.4 | 68.7 | 6,655 | 64.9 | 10.0 |  |
| 2015 | 67.1 | 1,123 | 67.4 | 1.7 | 4,920 | 60.5 | 7.3 | 3,470 | 82.7 | 4.6 | 26,668 | 13.4 | 39.7 | 45,588 | 15.9 | 67.9 | 7,452 | 84.6 | 11.1 |  |
| 2016 | 67.4 | 1,120 | 66.9 | 1.7 | 5,003 | 63.2 | 7.4 | 3,643 | 91.8 | 4.7 | 27,479 | 16.3 | 40.7 | 47,134 | 19.8 | 69.9 | 10,327 | 155.9 | 15.3 |  |
| 2017 | 67.5 | 1,244 | 85.4 | 1.8 | 5,146 | 67.9 | 7.6 | 3,823 | 101.3 | 4.7 | 27,889 | 17.2 | 41.3 | 47,053 | 19.6 | 69.7 | 11,493 | 184.8 | 17.0 |  |
| 2018 | 67.7 | 1,362 | 103.0 | 2.0 | 5,490 | 79.1 | 8.1 | 4,075 | 114.6 | 4.6 | 27,965 | 15.9 | 41.3 | 47,290 | 20.2 | 69.8 | 12,878 | 219.1 | 19.0 |  |
| 2019 | 67.9 | 1,385 | 106.4 | 2.0 | 5,593 | 82.5 | 8.2 | 4,201 | 121.2 | 4.6 | 28,057 | 15.4 | 41.3 | 47,874 | 21.7 | 70.5 | 15,175 | 276.0 | 22.4 |  |
