# Supplementary material for: Increased incidence of vertebral fractures in German adults from 2009 to 2019 and the analysis of secondary diagnoses, treatment, costs, and in-hospital mortality
Source: Sci Rep. 2023 Apr 28;13:6984. doi: 10.1038/s41598-023-31654-0 (PMC10147602; doi:10.1038/s41598-023-31654-0)
Supplement: Supplementary file 3 — Supplementary Information 3. [file 41598_2023_31654_MOESM3_ESM.docx]

| **G-DRG code** | **Cases [n]** | **Share of  cases** | **Mean Cost  per case [€]** | **Standard  deviation** | **Overall costs [€]** | **Share of overall costs** |
| --- | --- | --- | --- | --- | --- | --- |
| 801D | 71 | 0.1% | 9,104 | 3,747 | 646,384 | 0.11% |
| A09B | 48 | 0.0% | 67,782 | 16,714 | 3,253,536 | 0.55% |
| A09C | 97 | 0.1% | 56,786 | 14,527 | 5,508,242 | 0.93% |
| A13A | 58 | 0.1% | 50,936 | 13,353 | 2,954,288 | 0.50% |
| A13D | 157 | 0.1% | 25,215 | 8,142 | 3,958,755 | 0.67% |
| A90A | 625 | 0.6% | - | - | - | - |
| B61A | 75 | 0.1% | 12,495 | 7,087 | 937,125 | 0.16% |
| B61B | 390 | 0.4% | - | - | - | - |
| I06A | 98 | 0.1% | 26,257 | 8,051 | 2,573,186 | 0.44% |
| I06C | 192 | 0.2% | 17,623 | 5,243 | 3,383,616 | 0.57% |
| I08A | 68 | 0.1% | 18,515 | 5,928 | 1,259,020 | 0.21% |
| I08B | 257 | 0.2% | 14,358 | 5,567 | 3,690,006 | 0.63% |
| I08C | 96 | 0.1% | 11,119 | 3,611 | 1,067,424 | 0.18% |
| I08D | 420 | 0.4% | 8,825 | 3,334 | 3,706,500 | 0.63% |
| I08E | 285 | 0.3% | 8,173 | 2,314 | 2,329,305 | 0.40% |
| I08F | 651 | 0.6% | 5,981 | 1,695 | 3,893,631 | 0.66% |
| I09A | 1,087 | 1.0% | 21,747 | 7,537 | 23,638,989 | 4.01% |
| I09B | 337 | 0.3% | 19,336 | 5,795 | 6,516,232 | 1.11% |
| I09C | 681 | 0.6% | 17,283 | 5,116 | 11,769,723 | 2.00% |
| I09D | 999 | 1.0% | 15,880 | 5,319 | 15,864,120 | 2.69% |
| I09E | 3,925 | 3.7% | 13,194 | 3,892 | 51,786,450 | 8.79% |
| I09F | 7,785 | 7.4% | 10,160 | 3,169 | 79,095,600 | 13.42% |
| I09G | 1,030 | 1.0% | 8,828 | 2,323 | 9,092,840 | 1.54% |
| I09H | 2,487 | 2.4% | 6,791 | 2,245 | 16,889,217 | 2.87% |
| I09I | 9,050 | 8.6% | 5,180 | 1,642 | 46,879,000 | 7.96% |
| I10A | 800 | 0.8% | 21,747 | 7,537 | 17,397,600 | 2.95% |
| I10B | 1,722 | 1.6% | 19,336 | 5,795 | 33,296,592 | 5.65% |
| I10D | 110 | 0.1% | 4,601 | 1,420 | 506,110 | 0.09% |
| I10E | 185 | 0.2% | 3,757 | 1,132 | 695,045 | 0.12% |
| I10F | 657 | 0.6% | 3,249 | 1,026 | 2,134,593 | 0.36% |
| I21Z | 3,067 | 2.9% | 2,944 | 748 | 9,029,248 | 1.53% |
| I23A | 207 | 0.2% | 3,134 | 1,180 | 648,738 | 0.11% |
| I23B | 197 | 0.2% | 2,339 | 658 | 460,783 | 0.08% |
| I26Z | 351 | 0.3% | 33,290 | 11,974 | 11,684,790 | 1.98% |
| I34Z | 1580 | 1.5% | 10,853 | 2,328 | 17,147,740 | 2.91% |
| I40Z | 133 | 0.1% | - | - | - | - |
| I41Z | 10,825 | 10.3% | 5,878 | 1,317 | 63,629,350 | 10.80% |
| I68B | 4,465 | 4.3% | 4,689 | 2,334 | 20,936,385 | 3.55% |
| I68C | 7,190 | 6.8% | 3,297 | 1,604 | 23,705,430 | 4.02% |
| I68D | 35,000 | 33.3% | 1,708 | 636 | 59,780,000 | 10.15% |
| I68E | 5,069 | 4.8% | 719 | 262 | 3,644,611 | 0.62% |
| I87Z | 90 | 0.1% | 5,287 | 2,240 | 475,830 | 0.08% |
| W01B | 84 | 0.1% | 44,194 | 12,394 | 3,712,296 | 0.63% |
| W02A | 250 | 0.2% | 27,857 | 12,332 | 6,964,250 | 1.18% |
| W02B | 301 | 0.3% | 17,388 | 6,272 | 5,233,788 | 0.89% |
| W04A | 59 | 0.1% | 20,544 | 8,089 | 1,212,096 | 0.21% |
| W04B | 124 | 0.1% | 11,506 | 4,832 | 1,426,744 | 0.24% |
| W36Z | 63 | 0.1% | 52,694 | 15,433 | 3,319,722 | 0.56% |
| W61A | 57 | 0.1% | 6,363 | 3,369 | 362,691 | 0.06% |
| W61B | 218 | 0.2% | 5,083 | 2,634 | 1,108,094 | 0.19% |
