# Supplementary material for: Increased incidence of vertebral fractures in German adults from 2009 to 2019 and the analysis of secondary diagnoses, treatment, costs, and in-hospital mortality
Source: Sci Rep. 2023 Apr 28;13:6984. doi: 10.1038/s41598-023-31654-0 (PMC10147602; doi:10.1038/s41598-023-31654-0)
Supplement: Supplementary file 4 — Supplementary Legends. [file 41598_2023_31654_MOESM4_ESM.docx]

**Supplementary material**

**Supplementary material 1**: The distributions of vertebral fractures by anatomical location (A: Atlas & Axis; B: Subaxial cervical spine; C: Thoracic spine; D: Lumbar spine; E: Sacrum; F: Coccygis) for each year from 2009 to 2019 based on the Destatis data. Differentiation by age decade and sex.

**Supplementary material 2:** Total numbers of vertebral fractures depending on anatomical localization for each year from 2009 to 2019, relative change to 2009, and incidence per 100,000 inhabitants, based on the Destatis data.

**Supplementary material 3:** List of the 50 most commonly used G-DRG codes (at least 0.05% of all cases) for cases with vertebral fractures in 2019 with numbers of total cases, share of cases, costs per case [€], calculated sum costs per G-DRG [€] and shares of overall costs. Alphabetical sorting. Based on the InEK Data Browser.
